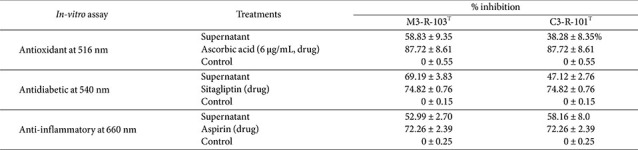# *Bifidobacterium intestinale* sp. nov., and *Blautia caeci* sp. nov., from the Human Gut Microbiome with Candidate Probiotic Potential

**DOI:** 10.4014/jmb.2603.03013

**Published:** 2026-05-25

**Authors:** Md Shamsuzzaman, Ram Hari Dahal, Jungmin Kim

**Affiliations:** 1 Untreatable Infectious Disease Institute, Kyungpook National University, Daegu 41944, Republic of Korea; 2 Department of Microbiology, School of Medicine, Kyungpook National University, Daegu 41944, Republic of Korea

**Keywords:** *Bifidobacterium intestinale* sp. nov., *Blautia caeci* sp. nov., Gut microbiome, Functional properties, Antioxidant, α-amylase inhibition, Anti-inflammatory

## Abstract

Two novel Gram-stain-positive, anaerobic, and non-motile bacterial strains, designated M3-R-103^T^ and C3-R-101^T^, were isolated from human faecal samples and subjected to comprehensive taxonomic characterization together with evaluation of selected functional properties. Phylogenetic analysis based on 16S rRNA gene sequences demonstrated that strain M3-R-103^T^ is affiliated with the genus *Bifidobacterium*, whereas strain C3-R-101^T^ belongs to the genus *Blautia*. Strain M3-R-103^T^ exhibited the highest 16S rRNA gene sequence similarity (98.9%) to *Bifidobacterium pseudocatenulatum* DSM 20438^T^, while strain C3-R-101^T^ showed 98.6% similarity to *Blautia hominis* KB1^T^. The draft genome sizes of strains M3-R-103^T^ and C3-R-101^T^ were 2.08 Mbp and 6.40 Mbp, respectively, with DNA G+C contents of 56.3 mol% and 46.7 mol%. Both strains demonstrated tolerance to simulated gastrointestinal stress conditions, including exposure to acidic pH (2.0) and bile salts (0.3%). *In vitro* assays revealed measurable antioxidant, α-amylase inhibitory, and anti-inflammatory activities. Specifically, DPPH radical scavenging activity ranged from 38.28 ± 8.35% to 58.83 ± 9.35%, α-amylase inhibition ranged from 47.12 ± 2.76% to 69.19 ± 3.83%, and inhibition of albumin denaturation ranged from 52.99 ± 2.70% to 58.16 ± 8.00%. Based on combined phylogenetic, genomic, and phenotypic evidence, strains M3-R-103^T^ and C3-R-101^T^ represent two novel species within their respective genera. We therefore propose the names *Bifidobacterium intestinale* sp. nov. (type strain M3-R-103^T^ = KCTC 25443^T^ = CGMCC 1.19415^T^) and *Blautia caeci* sp. nov. (type strain C3-R-101^T^ = KCTC 25440^T^ = CGMCC 1.17981^T^).

## Introduction

The genus *Bifidobacterium*, belonging to the family *Bifidobacteria*ceae within the phylum Actinomycetota, was first introduced by Orla-Jensen, with *Bifidobacterium bifidum* designated as the type species. Members of this genus are among the most extensively studied commensal bacteria of the human gastrointestinal tract and are frequently used as probiotics [1, 2]. Numerous studies have shown that *Bifidobacterium* species contribute to intestinal homeostasis through metabolic activities, competitive exclusion of pathogens, and modulation of host immune responses [3]. In addition, certain strains have been associated with a range of health-related effects, including anti-inflammatory, α-amylase inhibitory, antibacterial, and anticancer-associated activities, depending on strain identity and host context [4]. As of January 05, 2026, the genus *Bifidobacterium* comprises 119 validly published species and 20 subspecies, according to the List of Prokaryotic Names with Standing in Nomenclature (LPSN) database (https://lpsn.dsmz.de/genus/bifidobacterium). This continuously expanding taxonomy reflects both the ecological importance of *Bifidobacterium* and the ongoing discovery of previously unrecognized gut-associated diversity.

Similarly, the genus *Blautia* is classified within the family *Lachnospiraceae* of the phylum *Bacillota* and was delineated by Liu et al., with *Blautia coccoides* designated as the type species [5]. Species of *Blautia* are prevalent members of the adult human gut microbiota and are increasingly recognized for their potential roles in host physiology and intestinal homeostasis [6]. Several reports have linked *Blautia* abundance and strain-level composition with inflammatory bowel disease and metabolic disorders, suggesting that this genus may contribute to immune and metabolic regulation in the intestinal ecosystem [7, 8]. In addition, selected *Blautia* strains have been described to exert inhibitory effects against enteric pathogens and to influence gut microbial community structure through competitive interactions and metabolite-mediated mechanisms [6]. As of February 05, 2026, the genus *Blautia* comprises 31 recognized species according to LPSN (https://lpsn.dsmz.de/genus/blautia).

Species belonging to the genera *Bifidobacterium* and *Blautia* have been recovered from diverse habitats, including the human gastrointestinal tract, animal intestines, and fermented foods [1, 6]. Despite extensive advances in microbiome research, the taxonomic and functional diversity of gut-associated bacteria remains incompletely explored, and novel taxa continue to be recovered from healthy individuals across populations [9]. This is particularly relevant because strain-level differences can be substantial even among closely related taxa, leading to variation in metabolic potential, stress tolerance, antimicrobial activity, and interactions with host immune responses [10]. Therefore, the isolation and characterization of novel gut-derived bacteria remain important not only for expanding bacterial systematics but also for identifying candidate strains with probiotic-associated potential.

In parallel with taxonomic discovery, the evaluation of biosafety-related characteristics is increasingly emphasized in probiotic research. Even within genera that are generally considered beneficial, individual strains may differ in antibiotic susceptibility profiles, hemolytic activity, and other safety-relevant traits, which should be considered during strain selection and development [11, 12]. For this reason, polyphasic approaches that integrate taxonomy, genome-informed analyses, and functional screening provide a robust framework for evaluating gut-derived isolates for potential probiotic development.

In the present study, two previously uncharacterized anaerobic bacterial strains, designated M3-R-103^T^ and C3-R-101^T^, were isolated from faecal samples of healthy individuals. Phylogenetic and genomic analyses were conducted to determine their taxonomic positions within the genera *Bifidobacterium* and *Blautia*, respectively. In addition, their functional properties were evaluated using in vitro assays, including tolerance to simulated gastrointestinal conditions (acidic pH and bile salts) and assessment of antioxidant, α-amylase inhibitory, anti-inflammatory, antibacterial, and hemolytic activities. By integrating taxonomic characterization with functional and safety-related evaluation, this study expands the known diversity of human gut-associated bacteria and provides strain-level insights relevant to the identification of next-generation probiotic candidates.

## Materials and Methods

### Isolation of Strains

Strains M3-R-103^T^ and C3-R-101^T^ were isolated from healthy Korean human faecal samples as part of an effort to culture and characterize previously unrecognized gut commensal bacteria. Samples were serially diluted and plated on Brain Heart Infusion (BHI) agar supplemented with 5% defibrinated sheep blood under anaerobic conditions. After incubation at 37°C for 48 h, multiple morphologically distinct colonies were selected and purified by repeated streaking. Among the recovered anaerobic isolates, strains M3-R-103^T^ and C3-R-101^T^ were selected for further study based on preliminary 16S rRNA gene sequence analysis indicating potential taxonomic novelty. Pure cultures were temporarily maintained at 4°C and subsequently preserved in 20% (v/v) glycerol at -70°C for long-term storage. Although BHI supplemented with 5% sheep blood is a non-selective medium, strict anaerobic incubation conditions enabled the recovery of diverse gut-associated anaerobic bacteria, from which strains of interest were identified through colony selection and subsequent 16S rRNA gene analysis, including members of the genera *Bifidobacterium* and *Blautia*.

### 16S rRNA Gene Sequencing and Phylogenetic Analysis

Genomic DNA was extracted using the HiGene Genomic DNA Prep Kit (BIOFACT, Republic of Korea). The 16S rRNA gene was amplified using primers 27F and 1492R [13]. Sequence similarity searches were performed using BLAST (NCBI) and EzBioCloud [14]. Phylogenetic trees were reconstructed using MEGA-11 [15], with maximum-likelihood (ML) [16], neighbor-joining (NJ) [17], and maximum-parsimony (MP) [18] methods. Bootstrap support was calculated based on 1,000 replicates [19], and evolutionary distances were estimated using the Kimura two-parameter model [20].

### Genome Analysis

Genome sequencing for strains M3-R-103^T^ and C3-R-101^T^ was performed by Macrogen (Republic of Korea) using the Illumina HiSeq platform. Genome completeness was assessed using BUSCO (v3.0.2) [21] , and genome sequence quality and potential contamination were evaluated using the ContEst16S algorithm [22]. Gene prediction and functional annotation were performed using both the NCBI Prokaryotic Genome Annotation Pipeline (PGAP) and the Rapid Annotation Subsystem Technology (RAST) server [23], while additional functional categorization was assigned through the eggNOG 4.5 database [24]. Orthologous gene clustering and comparative genomic analyses were conducted using OrthoVenn3 [25]. Antibiotic resistance determinants were screened against the Comprehensive Antibiotic Resistance Database (CARD) [26], and biosynthetic gene clusters were identified using antiSMASH 7.0 [27]. ANI and dDDH values were calculated using established online tools [28, 29] to evaluate genomic relatedness. Phylogenomic analysis was performed using TYGS [30]. In addition, a core single-copy gene phylogeny based on 100 conserved genes was reconstructed using RAxML with 100 bootstrap replicates and visualized using iTOL [31]. Comparative whole-genome alignment was performed using progressive Mauve [32]. The genomic G+C content was calculated from the assembled genome sequences.

### Morphological, Physiological, and Biochemical Analysis

Cellular morphology was examined using transmission electron microscopy (TEM; JEM-1011, JEOL). Gram staining was performed using the Color Gram 2 kit (Sigma-Aldrich, USA). Anaerobic growth characteristics, motility, and catalase and oxidase activities were evaluated according to standard microbiological procedures [33]. Growth under aerobic and microaerophilic conditions was also assessed. Optimal physiological parameters, including temperature range, NaCl tolerance, and pH preference, were determined following established taxonomic protocols [34]. Phenotypic characterization was performed using API 20A, API ZYM, and API 50CH (bioMérieux).

### Evaluating Bacterial Resistance to Gastric and Intestinal Conditions

To evaluate gastrointestinal stress tolerance, acid and bile resistance of the bacterial strains were assessed under controlled laboratory conditions. Actively growing cultures (OD_600_ ≈ 1.0) were used for all experiments. Although BHI medium was used for initial isolation, MRS broth was employed as a standardized medium for functional assays, as it supported consistent growth of the strains under anaerobic conditions. For routine cultivation, Gifu Anaerobic Medium (GAM; code 05422) was also used. All assays were conducted under strict anaerobic conditions (90% N_2_, 5% CO_2_, 5% H_2_) at 37°C. General acid tolerance was assessed by incubating strains in MRS broth adjusted to pH 2.0–6.0 for 48 h. Short-term acid tolerance was further evaluated by exposing overnight-grown cultures to MRS broth adjusted to pH 2.0 and 4.0 (using 1 N HCl) for 4 h [35]. Bile salt resistance was evaluated by incubating cultures in MRS medium containing 0.3% and 0.5% (w/v) bile salts under anaerobic conditions. Growth was monitored after 3 h and 4 h of incubation by measuring optical density at 600 nm (OD_600_). The growth rate was calculated as % growth = (OD_600_ of treated culture / OD_600_ of control culture) × 100 [34]. To simulate gastric conditions, bacterial cultures were incubated in a pepsin solution (Sigma-Aldrich), prepared freshly according to the manufacturer’s instructions, with 0.85% NaCl at pH 2.5 for 4 h. Intestinal fluid conditions were evaluated by incubating bacteria with 0.3% bile salt (w/v) and 1 mg/mL pancreatin (Sigma-Aldrich), prepared freshly before use, at pH 8.0 for 6 h, and survival was determined based on viable cell counts. Survival percentage was calculated as % survival = (N/N_0_) × 100, where N_0_ represents the initial CFU/mL before treatment and N represents the CFU/mL after exposure [35].

### *In Vitro* Assessment of Antioxidant, α-amylase inhibitory, and Anti-inflammatory Activities of Bacterial Supernatant

Cell-free supernatants (CFS) were obtained from bacterial cultures grown in MRS broth under anaerobic conditions at 37°C until the late exponential phase. Cultures were centrifuged at 10,000 × g for 10 min, and the supernatants were collected and filtered through a 0.22 μm membrane to remove residual bacterial cells. The pH of the supernatants was adjusted to neutral (pH 7.0) to minimize acid-mediated effects. All cultures were standardized to the same initial optical density (OD_600_), and CFS was used at a fixed concentration of 10% (v/v) for all assays.

Antioxidant activity was determined using the DPPH radical scavenging assay. Briefly, CFS (10%, v/v) was mixed with DPPH solution in methanol and incubated at 37°C for 30 min in the dark. The decrease in absorbance was measured at 516 nm using a UV–visible spectrophotometer [36]. Ascorbic acid (6 μg/mL) was used as the positive control. Negative control samples consisted of DPPH solution without bacterial supernatant (replaced with sterile MRS broth or methanol as appropriate).

α-Amylase inhibitory activity was assessed using a modified DNSA method. Briefly, 500 μL of CFS (10%, v/v) was mixed with 500 μL of 0.02 M sodium phosphate buffer (pH 6.9 containing 0.006 M NaCl) with porcine pancreatic α-amylase (0.5 mg/mL) and incubated at 25°C for 10 min. Subsequently, 500 μL of 1% starch solution prepared in the same buffer was added, and the mixture was incubated for an additional 10 min. The reaction was terminated by adding 1.0 mL of dinitrosalicylic acid (DNSA) reagent, followed by boiling for 5 min. After cooling to room temperature, 10 mL of distilled water was added, and absorbance was measured at 540 nm [37]. Sitagliptin was used as the positive control, prepared under the same assay conditions. Negative control samples consisted of reaction mixtures without bacterial supernatant (replaced with sterile MRS broth or buffer).

Anti-inflammatory activity was evaluated using an albumin denaturation inhibition assay. Briefly, 500 μL of CFS (10%, v/v) was mixed with an equal volume of 1% (w/v) bovine serum albumin solution. The reaction mixture was incubated at room temperature for 20 min, followed by heating at 51°C for 20 min. After cooling, turbidity was measured at 660 nm using a UV–visible spectrophotometer [37]. Aspirin was used as the positive control, prepared under identical conditions. Negative control samples consisted of assay mixtures without bacterial supernatant (replaced with sterile MRS broth or buffer).

A separate growth inhibition assay was performed using a bacterial suspension and cell-free supernatant incubated at 37°C for 24 h, with OD measured at 630 nm. The inhibition percentage for all assays was calculated as [(Ac − As) / Ac] × 100, where Ac represents the absorbance of the control and As represents the absorbance of the sample [38]. These assays were employed as preliminary in vitro screening tools to evaluate functional properties associated with gut-derived bacterial metabolites and do not imply in vivo efficacy. All experiments were performed in triplicate (n = 3), and results are presented as mean ± standard deviation (SD).

### Hemolytic Activity

Hemolytic activity was assessed by observing growth on blood agar (BHI with 5% defibrinated sheep blood) at 37°C for 48 h. α- and γ-hemolysis were considered nonhemolytic; β-hemolysis indicated hemolysis [34]. *Segatella intestinalis* B2-R-102^T^ was used as a non-hemolytic (negative) control, while *Cutibacterium acnes* KB112 was used as a hemolytic (positive) control.

### Antimicrobial Susceptibility Testing

Antimicrobial susceptibility was determined using the broth microdilution method in accordance with CLSI M11 (9th edition) guidelines for anaerobic bacteria. All assays were conducted under strict anaerobic conditions, and the test medium supported adequate growth of the strains without additional supplementation. Mid-log phase cultures were adjusted to approximately 5 × 10^5^ CFU/mL and inoculated into 96-well microtiter plates containing two-fold serial dilutions of the tested antibiotics, including cefoxitin, ampicillin, piperacillin/tazobactam, imipenem, erythromycin, tetracycline, and vancomycin. Plates were incubated under anaerobic conditions at 37°C for 48 h. The minimum inhibitory concentration (MIC) was defined as the lowest antibiotic concentration that completely inhibited visible growth. All experiments were performed in triplicate. Where applicable, MIC values were interpreted according to CLSI guidelines for anaerobic bacteria; however, for antibiotics without established genus-specific breakpoints, results were reported without categorical interpretation [39].

### Antimicrobial Activity of Cell-Free Supernatants

The antimicrobial activity of cell-free supernatants (CFS) was evaluated using a broth-based co-incubation growth inhibition assay [40]. CFS was obtained by centrifugation of overnight cultures followed by filtration (0.22 μm) to remove bacterial cells. Indicator pathogens were grown to mid-log phase and inoculated into fresh broth in the presence or absence of CFS (10%, v/v). Cultures were incubated at 37°C for 24 h, and bacterial growth was monitored by measuring optical density at 600 nm (OD_600_). Growth inhibition was calculated as % inhibition = [(OD_600_ of control − OD_600_ of treated culture) / OD_600_ of control] × 100. The control consisted of pathogen cultures grown without CFS under identical conditions. All experiments were performed in triplicate.

### Statistical Analysis

All experiments were performed in triplicate (n = 3), and results are presented as mean ± standard deviation (SD). Data are reported descriptively, and no formal statistical significance testing was performed.

## Results

### Biochemical and Morphological Characterization

Strains M3-R-103^T^ and C3-R-101^T^ were Gram-stain-positive, strictly anaerobic, non-spore-forming, and non-motile rod-shaped bacteria, with no growth observed under aerobic or microaerophilic conditions. Both strains exhibited robust growth on BHI agar supplemented with 5% defibrinated sheep blood, forming circular, convex, and smooth colonies after 48 h of anaerobic incubation at 37°C. Strain M3-R-103^T^ produced opaque to slightly creamy colonies, whereas strain C3-R-101^T^ formed slightly translucent colonies with comparable morphology. Growth profiling showed that strain M3-R-103^T^ grew at temperatures ranging from 30 to 42°C, while strain C3-R-101^T^ exhibited a broader temperature range of 20 to 42°C. Both strains displayed broad pH tolerance, with optimal growth between pH 5.5 and 8.0 and survival across pH 2.0–10.0, indicating substantial acid–alkali resilience. A detailed comparison of morphological and biochemical characteristics is provided in Table 1 and Fig. S1.

### Phylogenetic Analysis

**Fig. 1:**
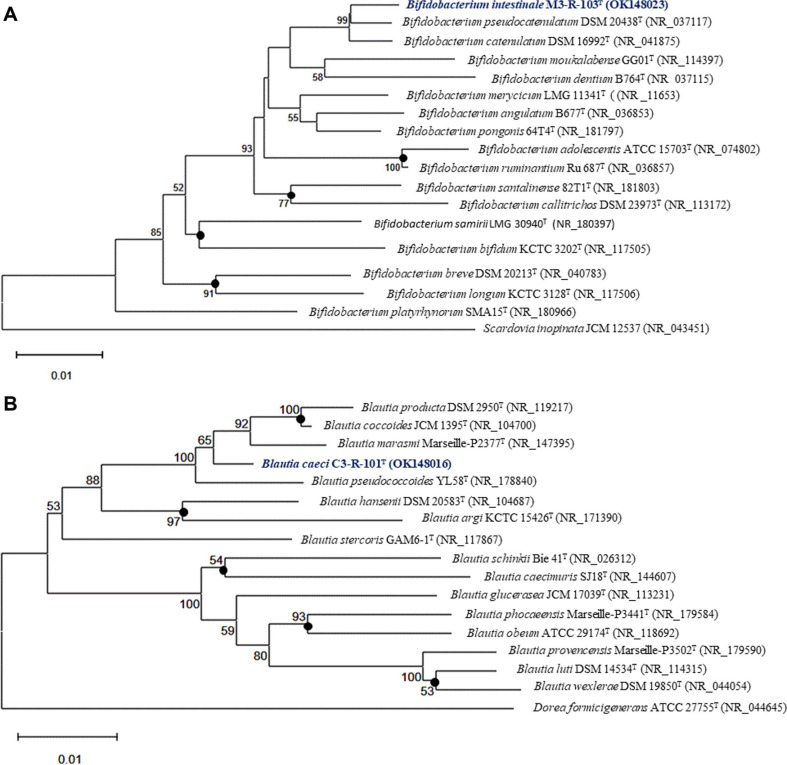
Maximum likelihood (ML) phylogenetic trees based on nearly complete 16S rRNA gene sequences showing the taxonomic positions of strains M3-R-103^T^ and C3-R-101^T^ within the genera *Bifidobacterium* (A) and *Blautia* (B) respectively. Bootstrap values based on 1000 replicates are shown at branch nodes, with only values ≥50% indicated. Nodes consistently recovered by neighbor-joining, maximum-likelihood, and maximum-parsimony methods are marked with filled circles. *Scardovia inopinata* JCM 12537^T^ (NR_043451) and *Dorea formicigenerans* ATCC 27755^T^ (NR_044645) were used as outgroup taxa for panels A and B, respectively. Nucleotide sequence accession numbers are given in parentheses. The scale bar represents 0.01 substitutions per nucleotide position.

Nearly full-length 16S rRNA gene sequences were obtained for strains M3-R-103^T^ and C3-R-101^T^, yielding amplicons of 1,447 bp and 1,470 bp, respectively, using universal bacterial primers 27F and 1492R. These sequences were used for preliminary taxonomic placement and subsequently validated against the corresponding whole-genome assemblies. Genome analysis revealed that strain M3-R-103^T^ harbors four rRNA operons, whereas strain C3-R-101^T^ contains a single complete 16S rRNA gene copy. In both strains, the genome-derived 16S rRNA gene sequences were identical to the PCR-amplified sequences, confirming sequence accuracy and excluding intragenomic heterogeneity. Comparative 16S rRNA gene sequence analysis showed that strain M3-R-103^T^ is affiliated with the genus *Bifidobacterium*, exhibiting its highest sequence similarity (98.9%) to *Bifidobacterium pseudocatenulatum* DSM 20438^T^. In contrast, strain C3-R-101^T^ clustered within the genus *Blautia*, sharing 98.6% sequence similarity with *Blautia hominis* KB1^T^. Both strains displayed sequence similarity values below the commonly accepted species-level threshold (98.7–99.0%), indicating that they represent distinct taxa (Table S1). Phylogenetic trees reconstructed using maximum-likelihood, neighbour-joining, and maximum-parsimony methods consistently supported these affiliations. In all tree topologies, strain M3-R-103^T^ formed a well-supported, independent lineage within the *Bifidobacterium* clade, clearly separated from *B. pseudocatenulatum* and related taxa. Similarly, strain C3-R-101^T^ formed a distinct branch within the genus *Blautia*, separated from *B. hominis* and closely related species. The congruent topologies obtained using multiple phylogenetic methods further support the phylogenetic distinctiveness of both strains (Fig. 1; Fig. S2).

### Genome Analysis

Whole-genome sequencing of strains M3-R-103^T^ and C3-R-101^T^ yielded high-quality draft genomes with sizes of 2,080,619 bp and 6,401,851 bp, respectively. The genomic G+C contents were 56.3% for strain M3-R-103^T^ and 46.7% for strain C3-R-101^T^, values consistent with those reported for members of the genera *Bifidobacterium* and *Blautia*, respectively. Genome quality assessment confirmed the absence of detectable contamination, and both assemblies showed high sequencing coverage (>150×), supporting their completeness and reliability (Table S2).

Functional annotation using the RAST pipeline identified 518 subsystem features in strain M3-R-103^T^ and 1,106 subsystem features in strain C3-R-101^T^ (Figs. S3), reflecting substantial differences in genome size and metabolic potential between the two strains. Comparative genome-based analyses further supported the taxonomic distinctiveness of both strains. Average nucleotide identity (ANI) and digital DNA–DNA hybridization (dDDH) values between strain M3-R-103^T^ and its closest relatives within the genus *Bifidobacterium* ranged from 76.5–93.6% and 24.1–54.5%, respectively, with the highest relatedness observed against *Bifidobacterium pseudocatenulatum* DSM 20438^T^ (ANI 92.1%, dDDH 44.9%). Similarly, strain C3-R-101^T^ exhibited ANI values of 72.8–84.8% and dDDH values of 22.8–30.0% relative to closely related *Blautia* species, showing the greatest similarity to *Blautia hominis* KB1^T^ (ANI 83.9%, dDDH 29.6%). All values remained well below the accepted species delineation thresholds (ANI <95%; dDDH <70%), clearly indicating that both strains represent distinct genomic species (Table S3).

**Fig. 2:**
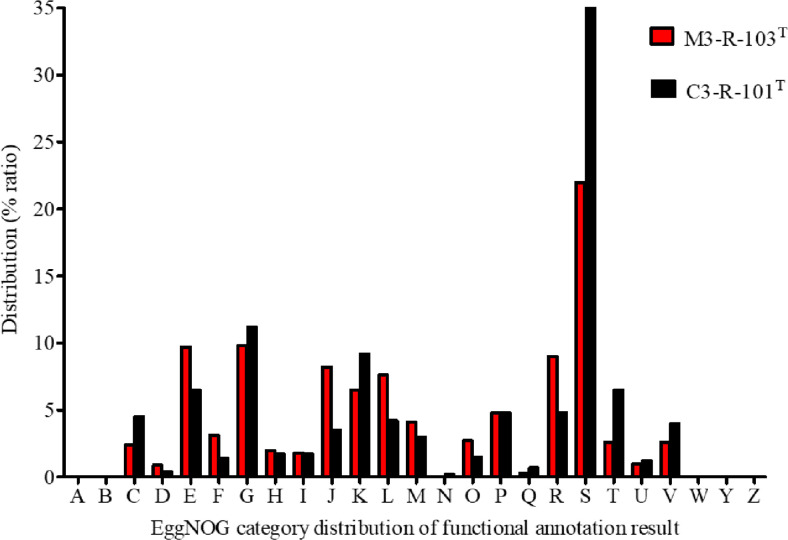
Distribution of Cluster of Orthologous Group (COG) functional categories of the genome of strains M3-R-103^T^ and C3-R-101^T^, where (A) RNA processing and modification. (**B**) Chromatin structure and dynamics, (**C**) Energy production and conversion, (**D**) Cell cycle control, cell division, chromosome partitioning, (**E**) Amino acid transport and metabolism, (**F**) Nucleotide transport and metabolism, (**G**) Carbohydrate transport and metabolism, (**H**) Coenzyme transport and metabolism, (**I**) Lipid transport and metabolism, (**J**) Translation, ribosomal structure and biogenesis, (**K**) Transcription, (**L**) Replication, recombination and repair, (**M**) Cell wall/membrane/envelope biogenesis, (**N**) Cell motility, (**O**) Posttranslational modification, protein turnover, chaperones, (**P**) Inorganic ion transport and metabolism, (**Q**) Secondary metabolites biosynthesis transport and catabolism, (**R**) General function prediction only, (**S**) Function unknown, (**T**) Signal transduction mechanisms, (**U**) Intracellular trafficking, secretion, and vesicular transport, (**V**) Defence mechanisms, (**W**) Extracellular structures, (**Y**) Nuclear structure, (**Z**) Cytoskeleton

A phylogenomic tree based on the concatenated alignment of 100 single-copy core genes was reconstructed using RAxML with 100 bootstrap replicates. The resulting topology clearly placed strain M3-R-103^T^ within the genus *Bifidobacterium* and strain C3-R-101^T^ within the genus *Blautia*, consistent with TYGS analysis and 16S rRNA gene phylogeny. Both strains formed distinct branches separate from their closest type strains, supporting their recognition as novel species (Fig. S4). Orthologous gene cluster analysis conducted using the EggNOG pipeline assigned 2,154 genes from strain M3-R-103^T^ and 5,824 genes from strain C3-R-101^T^ to 25 COG functional categories. A considerable proportion of genes were classified under the “function unknown” category [S], accounting for 22% of the genes in strain M3-R-103^T^ and 35% in strain C3-R-101^T^. Among the annotated functional categories, genes involved in energy production and conversion [C], amino acid transport and metabolism [E], and carbohydrate transport and metabolism [G] were highly represented in both genomes. Additional abundant categories included those associated with transcription [K], translation and ribosomal structure [J], and replication, recombination, and repair, highlighting conserved core cellular and metabolic functions (Fig. 2).

Genome-based screening for antimicrobial resistance and virulence determinants detected a limited number of resistance-associated genes in strain C3-R-101^T^, whereas no such genes were identified in strain M3-R-103^T^. Specifically, the genome of strain C3-R-101^T^ contained predicted tetracycline and macrolide resistance determinants, including *tet(O)*, *msr(D)*, *mef(A)*, and *erm(B)*, consistent with WGS-based predictions of resistance to tetracyclines and macrolides (Tables S3 and S4). No resistance was predicted for other antimicrobial classes examined, including β-lactams, aminoglycosides, quinolones, polymyxins, fosfomycin, and folate pathway antagonists. Independent analysis using the Comprehensive Antibiotic Resistance Database (CARD) did not identify any high-confidence, curated antimicrobial resistance genes in either strain, suggesting that several predicted hits may represent low-confidence annotations or non-functional homologs (Figs. S5). In addition, no plasmid-associated replicons were detected in either genome.

BGC analysis revealed the presence of multiple putative secondary metabolite–associated regions in strain C3-R-101^T^. Specifically, the genome of C3-R-101^T^ harbored gene clusters predicted to encode a nonribosomal peptide synthetase (NRPS), a cyclic lactone–type autoinducer, a ranthipeptide, and an RRE-containing cluster. In contrast, no biosynthetic gene clusters were detected in the genome of M3-R-103^T^, indicating a comparatively limited potential for secondary metabolite production in this strain (Fig. S6). Comparative whole-genome alignment using progressive MAUVE revealed that more than 95% of the genomes of strain M3-R-103^T^ and *Bifidobacterium pseudocatenulatum* DSM 20438^T^, as well as strain C3-R-101^T^ and *Blautia hominis* KB1^T^, were conserved, indicating substantial synteny and a shared evolutionary backbone. Several strain-specific genomic regions, observed as discontinuous segments in the alignments, highlighted unique genetic features and potential functional adaptations (Fig. S7).

### *In vitro* Probiotic Characteristics

Strains M3-R-103^T^ and C3-R-101^T^ demonstrated tolerance to simulated gastrointestinal stress conditions. Both strains retained viability following 4 h exposure to acidic environments (pH 2.0–4.0), with values ranging from 65.16–92.77% for M3-R-103^T^ and 80.24–88.70% for C3-R-101^T^ (Table 2). Under simulated gastric conditions containing pepsin (pH 2.0–3.0), both strains exhibited values exceeding 100% in several conditions, indicating that cell proliferation occurred under these conditions rather than survival alone. Survival in simulated intestinal fluid supplemented with pancreatin and bile salts at pH 8.0 remained high after 6 h, with M3-R-103^T^ showing numerically higher values than C3-R-101^T^. Both strains also exhibited bile salt tolerance, retaining ≥86% relative growth at 0.3% (w/v) oxgall and ≥68% at 0.5% (w/v) after exposure (Table 3). These observations are based on *in vitro* assays.

### Hemolytic Activity

Hemolytic activity assays revealed that strains M3-R-103^T^ and C3-R-101^T^ exhibited no hemolysis on blood agar after 48 h of anaerobic incubation, indicating a γ-hemolytic (non-hemolytic) phenotype (Fig. S8). The absence of hemolytic activity supports the biosafety profile of both strains and is consistent with established safety criteria for probiotic candidate microorganisms.

### *In vitro* Functional Activities

The *in vitro* antioxidant, α-amylase inhibitory, and anti-inflammatory activities of strains M3-R-103^T^ and C3-R-101^T^ are summarized in Table 4. Both strains exhibited measurable antioxidant activity, with strain M3-R-103^T^ showing a higher DPPH radical scavenging capacity (58.83 ± 9.35%) than strain C3-R-101^T^ (38.28 ± 19.31%), although these values were lower than that of the positive control, ascorbic acid (87.72 ± 8.61%). In the α-amylase inhibition assay, both strains demonstrated measurable α-amylase inhibitory activity, with inhibition rates of 69.19 ± 3.83% for M3-R-103^T^ and 47.12 ± 2.76% for C3-R-101^T^, approaching the activity observed for sitagliptin (74.82 ± 0.76%). Anti-inflammatory activity, assessed by inhibition of protein (albumin) denaturation, was also detected in both strains, with inhibition values of 52.99 ± 2.70% for M3-R-103^T^ and 58.16 ± 8.00% for C3-R-101^T^, which were lower than that of aspirin (72.26 ± 2.39%). Collectively, these results indicate that both strains exhibit strain-dependent in vitro functional activities. These observations are based on in vitro assays and do not imply *in vivo* efficacy.

### Antimicrobial Activity of Cell-Free Supernatants

The antimicrobial activity of cell-free supernatants (CFS) obtained from strains M3-R-103^T^ and C3-R-101^T^ was evaluated against a panel of Gram-negative and Gram-positive indicator pathogens using a co-incubation growth inhibition assay (Table S4). Both strains exhibited strain- and pathogen-dependent inhibitory effects. The CFS of strain M3-R-103^T^ showed notable growth inhibition against *Staphylococcus felis* ATCC 49168 (65.37 ± 1.10%) and *Acinetobacter junii* strain 16 (70.41 ± 7.84%), whereas moderate inhibition was observed against *Staphylococcus pseudintermedius* ATCC 49051 (39.33 ± 3.00%) and *Staphylococcus epidermidis* ATCC 12228 (43.46 ± 1.71%). Similarly, the CFS of strain C3-R-101^T^ exhibited inhibitory activity against *A. junii* strain 16 (77.09 ± 4.81%), *S. felis* ATCC 49168 (63.37 ± 0.11%), and *S. epidermidis* ATCC 12228 (52.67 ± 0.17%). In contrast, neither strain demonstrated inhibitory activity against *Escherichia coli* ATCC 25922 or *Acinetobacter baumannii* ATCC 17978; instead, negative inhibition values were recorded, indicating no growth suppression under the tested conditions. Only low-level inhibition was observed against *Salmonella enteritidis* PT4 and *Staphylococcus schleiferi* ATCC 43808. These findings indicate that the antimicrobial effects of both strains are selective and likely mediated by secreted metabolites present in the CFS, rather than broad-spectrum antibacterial activity.

### Antimicrobial Susceptibility Profiles

The antimicrobial susceptibility profiles of strains M3-R-103^T^ and C3-R-101^T^ were determined by broth microdilution under anaerobic conditions, and MIC values are summarized in Table S5. Both strains exhibited low MIC values for β-lactam antibiotics, including cefoxitin (≤2–4 μg/mL), ampicillin (≤0.125 μg/mL), piperacillin/tazobactam (≤2–4 μg/mL), and imipenem (≤0.5–1 μg/mL). Strain M3-R-103^T^ showed low MIC values for erythromycin (≤0.25 μg/mL) and tetracycline (≤2 μg/mL), whereas strain C3-R-101^T^ displayed a higher erythromycin MIC (1 μg/mL) and an elevated tetracycline MIC (≥16 μg/mL). Both strains exhibited high MIC values for vancomycin (≥16 μg/mL). Differences in tetracycline and macrolide MIC values between the two strains were consistent with the genomic identification of resistance-associated determinants in strain C3-R-101^T^.

## Discussion

The present study describes the isolation and comprehensive characterization of two gut-derived anaerobic bacterial strains, M3-R-103^T^ and C3-R-101^T^, recovered from healthy human faecal samples and representing novel taxa within the genera *Bifidobacterium* and *Blautia*, respectively [1,6]. Both genera are well-established components of the human gut microbiota and have been widely associated with host metabolic regulation, immune modulation, and maintenance of intestinal homeostasis [41]. By integrating phenotypic, phylogenetic, genomic, and *in vitro* functional analyses, this work expands the known diversity of human-associated Actinomycetota and *Bacillota* and provides strain-level insights relevant to probiotic screening and biosafety evaluation.

Phenotypically, both strains displayed characteristics typical of intestinal commensals, including strict anaerobiosis, Gram-stain-positive cell structure, and non-motile rod-shaped morphology. Their ability to grow over broad temperature and pH ranges and to survive across a wide pH spectrum (pH 2.0–10.0) reflects adaptation to the dynamic physicochemical conditions of the gastrointestinal tract. Similar stress-tolerance profiles have been reported for well-characterized probiotic *Bifidobacterium* species, such as *B. longum*, *B. breve*, and *B. pseudocatenulatum*, which are capable of surviving transient exposure to gastric acidity and bile salts during intestinal passage [41, 42]. Members of the genus *Blautia* have likewise been reported to persist under variable gut conditions and are frequently detected as stable components of the adult gut microbiota [43]. While such physiological flexibility supports gastrointestinal survival, it should be interpreted as an adaptive trait rather than evidence of stable colonization or direct clinical benefit.

Phylogenetic analysis based on near-complete 16S rRNA gene sequences consistently placed strain M3-R-103^T^ within the genus *Bifidobacterium* and strain C3-R-101^T^ within the genus *Blautia*. In both cases, sequence similarities to their closest validly published relatives (*Bifidobacterium pseudocatenulatum* DSM 20438^T^ and *Blautia hominis* KB1^T^, respectively) remained below the commonly accepted species-level threshold [28, 44]. The congruent topologies obtained using multiple phylogenetic reconstruction methods further support the robustness of these taxonomic placements and minimize the likelihood that the observed separation reflects methodological artifacts or intragenomic heterogeneity [45].

Genome-based analyses provided strong support for the taxonomic novelty of both strains. Whole-genome comparisons revealed average nucleotide identity and digital DNA–DNA hybridization values well below recognized species demarcation thresholds, reinforcing their classification as distinct genomic species [29, 46]. Additional genome-based metrics such as average amino acid identity (AAI) may further refine phylogenomic resolution, although current ANI- and dDDH-based analyses provide robust support for species delineation. Genome-resolved taxonomy is now regarded as the gold standard for prokaryotic species delineation and has been widely applied in recent descriptions of novel gut-associated *Bifidobacterium* and *Blautia* taxa [47]. Comparative whole-genome alignments revealed substantial synteny with related species, suggesting conserved genomic organization, whereas discrete strain-specific regions likely reflect lineage-specific adaptations or microevolutionary divergence rather than extensive genome rearrangements.

Functional genome annotation revealed notable differences in metabolic potential between the two strains, largely attributable to their contrasting genome sizes. Both genomes were enriched in genes associated with carbohydrate transport and metabolism, amino acid utilization, and energy production, which are characteristic features of gut commensals involved in fermentation and cross-feeding interactions within the intestinal ecosystem [48, 49]. Such metabolic capabilities are central to the ecological roles of *Bifidobacterium* and *Blautia* species in maintaining intestinal homeostasis and metabolic balance [50]. Comparative genomic analysis further revealed several strain-specific features relative to their closest phylogenetic relatives. While most core functional categories were conserved, the presence of unique gene clusters suggests potential niche specialization and functional divergence at the strain level. The substantial proportion of genes annotated as hypothetical further highlights the incomplete functional understanding of strain-level diversity within these genera. However, further functional validation is required to confirm the biological significance of these genomic differences. Biosynthetic gene cluster (BGC) analysis further revealed the presence of putative secondary metabolite-associated gene clusters in both genomes. Although the predicted clusters were limited in number, they may potentially encode bioactive compounds such as bacteriocin-like molecules or other small metabolites that could contribute to microbial competition and ecological fitness within the gut environment. However, the exact products and functional roles of these clusters remain uncertain and require experimental validation.

From a safety perspective, both strains exhibited non-hemolytic (γ-hemolytic) phenotypes, fulfilling a fundamental criterion for probiotic candidacy [48]. Whole-genome screening detected no plasmids or confirmed virulence determinants in either strain. Although resistance-associated genes were predicted in strain C3-R-101^T^ by one annotation pipeline, these predictions were not supported by independent CARD analysis, suggesting that they likely represent low-confidence annotations or non-functional homologs. Importantly, no mobile genetic elements, such as plasmids, transposons, or integrative conjugative elements, were detected in association with these genes, indicating a low potential for horizontal gene transfer. Chromosomally encoded resistance determinants without mobility have been reported in several gut commensals and are generally not considered a safety concern when they are non-transferable and lack phenotypic expression. These observations are consistent with current EFSA and FAO/WHO guidelines, which emphasize that probiotic safety assessment should consider gene mobility and transferability rather than the mere presence of resistance-related sequences [34, 35]. Therefore, the detected resistance signals in strain C3-R-101^T^ are unlikely to pose a significant safety risk, although further functional validation may be required.

Both strains demonstrated notable tolerance to simulated gastrointestinal stress conditions, including exposure to acidic pH, bile salts, gastric enzymes, and intestinal fluids. Comparable levels of stress tolerance have been reported for established probiotic *Bifidobacterium* strains and are considered prerequisites for transient survival during gastrointestinal transit [2, 42]. However, it is important to recognize that such *in vitro* models represent simplified systems and cannot fully replicate the complexity of host–microbe interactions *in vivo*; therefore, these findings should be interpreted as indicators of physiological robustness rather than predictors of *in vivo* efficacy [48].

*In vitro* functional assays revealed strain-dependent antioxidant, α-amylase inhibitory, anti-inflammatory, and selective antimicrobial activities. Although these effects were consistently lower than those of pharmaceutical reference compounds, their magnitudes fall within ranges previously reported for gut-associated commensals and probiotic candidates, including members of *Bifidobacterium* and *Blautia* [34, 51]. These activities likely reflect indirect contributions to host metabolic and immune homeostasis rather than direct therapeutic effects. The selective antimicrobial activity observed for cell-free supernatants, primarily against Gram-positive indicator strains, suggests that secreted metabolites may influence microbial community balance in a context-dependent manner, a pattern commonly observed among commensal gut bacteria rather than broad-spectrum pathogen suppression [52].

In summary, strains M3-R-103^T^ and C3-R-101^T^ represent two novel species within the genera *Bifidobacterium* and *Blautia*, respectively, supported by robust phenotypic, phylogenetic, and genomic evidence. Their physiological resilience, favorable safety profiles, and moderate in vitro functional activities are consistent with traits reported for other beneficial human gut commensals. The DPPH scavenging, α-amylase inhibition, and albumin denaturation assays are indirect and non-specific screening methods that do not fully reflect in vivo physiological conditions. These assays primarily indicate the presence of bioactive metabolites and provide only preliminary functional insights. Therefore, the observed activities should be interpreted cautiously, and further validation using cell-based models and in vivo studies is required. While these findings support their inclusion among candidate beneficial gut bacteria, further mechanistic investigations are required to clarify their ecological roles and potential health implications. Furthermore, chemotaxonomic analyses such as fatty acid and polar lipid profiling were not performed in this study; however, genome-based and phylogenomic analyses provide robust support for the classification of the proposed strains.

## Conclusion

In this study, two gut-derived anaerobic bacterial strains, M3-R-103^T^ and C3-R-101^T^, were isolated from healthy human faecal samples and characterized using a comprehensive polyphasic approach. Phylogenetic and genome-based analyses demonstrated that these strains represent two novel species within the genera *Bifidobacterium* and *Blautia*, supported by 16S rRNA gene sequence analysis and whole-genome comparisons, including average nucleotide identity and digital DNA–DNA hybridization. Both strains exhibited physiological traits consistent with gastrointestinal adaptation, including tolerance to acidic conditions, bile salts, and simulated gastric and intestinal environments. Safety-related assessments revealed non-hemolytic phenotypes and the absence of plasmids, confirmed virulence determinants, and high-confidence antimicrobial resistance genes. Functional genome annotation indicated metabolic capacities typical of gut-associated commensals, particularly in carbohydrate and amino acid metabolism. *In vitro* functional assays further revealed strain-dependent antioxidant, α-amylase inhibitory, anti-inflammatory, and selective antimicrobial activities. While these observations indicate functional potential, they are moderate in magnitude and derived from in vitro assays, and therefore do not imply clinical efficacy. Overall, this study expands the catalog of human gut-associated bacteria and provides a foundation for future investigations into the ecological roles and functional relevance of *Bifidobacterium* and *Blautia* species in the human gastrointestinal tract.

## Species Protologue

### Description of *Bifidobacterium intestinale* sp. nov.

*Bifidobacterium intestinale* (in.tes.ti.na’le. N.L. neut. adj. *intestinale*, pertaining to the gut).

Cells are Gram-stain-positive, non-spore-forming, non-motile rods. Colonies on brain heart infusion agar supplemented with 5% defibrinated sheep blood are circular, smooth, convex, with entire margins, and approximately 1 mm in diameter after 48 h of anaerobic incubation at 37°C. Growth occurs at 30–42°C (optimum 37°C) and at pH 5.5–8.0 (optimum pH 7.0–7.5). Cells exhibit tolerance to acidic conditions and can survive short-term exposure to pH 2.0. The type strain is catalase-negative, indole-negative, urease-negative, and negative for glutamic acid decarboxylase. Gelatin is not hydrolysed, whereas esculin is hydrolysed. Acid is produced from L-arabinose, D-ribose, D-xylose, methyl-β-D-xylopyranoside, D-galactose, D-glucose, D-fructose, D-mannose, L-sorbose, D-mannitol, D-sorbitol, methyl-α-D-mannopyranoside, methyl-α-D-glucopyranoside, N-acetylglucosamine, amygdalin, arbutin, salicin, D-cellobiose, D-maltose, D-lactose, D-melibiose, D-sucrose, inulin, D-melezitose, D-raffinose, starch, glycogen, gentiobiose, D-turanose, and potassium gluconate. Acid is weakly produced from xylitol. Acid is not produced from glycerol, erythritol, D-arabinose, L-xylose, D-adonitol, L-rhamnose, dulcitol, inositol, D-trehalose, D-lyxose, D-tagatose, D-fucose, L-fucose, D-arabitol, L-arabitol, potassium 2-ketogluconate, or potassium 5-ketogluconate. Enzyme activities are positive for esterase (C4), esterase lipase (C8), leucine arylamidase, valine arylamidase, cystine arylamidase, acid phosphatase, naphthol-AS-BI-phosphohydrolase, α-galactosidase, β-galactosidase, α-glucosidase, and β-glucosidase; weakly positive for trypsin and α-chymotrypsin; and negative for alkaline phosphatase, lipase (C14), β-glucuronidase, N-acetyl-β-glucosaminidase, α-mannosidase, and α-fucosidase.

The DNA G+C content of the type strain is 56.3 mol% and the genome size is 2.08 Mb. The type strain, M3-R-103^T^ (= KCTC 25443^T^ = CGMCC 1.19415^T^), was isolated from human faeces. The GenBank accession numbers for the 16S rRNA gene sequence and the genome sequence are OK148023 and JAMOKR000000000, respectively.

### Description of *Blautia caeci* sp. nov.

*Blautia caeci* (cae’ci. N.L. gen. n. *caeci*, of the caecum)

Cells are Gram-stain-positive, strictly anaerobic, non-motile, non-spore-forming rods, approximately 1–1.5 μm in length. Colonies grown on brain heart infusion agar supplemented with 5% (v/v) defibrinated sheep blood are circular, umbonate, slightly glistening, white, with translucent edges, and reach approximately 1 mm in diameter after 48 h of anaerobic incubation at 37°C. Growth occurs at 20–42°C (optimum 37°C) and at pH 5.5–8.0 (optimum pH 7.0). Cells are tolerant to acidic conditions and can survive short-term exposure to low pH. The type strain is catalase-negative, indole-negative, urease-negative, and negative for glutamic acid decarboxylase. Gelatin is not hydrolysed, whereas esculin is hydrolysed. Acid is produced from D-arabinose, L-arabinose, D-ribose, D-xylose, D-lyxose, methyl-β-D-xylopyranoside, D-galactose, D-glucose, D-fructose, D-mannose, L-sorbose, L-rhamnose, inositol, D-mannitol, methyl-α-D-mannopyranoside, methyl-α-D-glucopyranoside, amygdalin, arbutin, salicin, D-cellobiose, D-maltose, D-lactose, D-melibiose, D-sucrose, D-trehalose, D-melezitose, D-raffinose, gentiobiose, D-turanose, L-arabitol, and potassium gluconate. Acid is weakly produced from xylitol and D-arabitol. Acid is not produced from glycerol, erythritol, L-xylose, D-adonitol, dulcitol, D-sorbitol, N-acetylglucosamine, inulin, starch, glycogen, D-tagatose, D-fucose, L-fucose, potassium 2-ketogluconate, or potassium 5-ketogluconate. Enzyme activities are positive for alkaline phosphatase, esterase (C4), esterase lipase (C8), lipase (C14), leucine arylamidase, valine arylamidase, cystine arylamidase, acid phosphatase, naphthol-AS-BI-phosphohydrolase, α-galactosidase, β-galactosidase, β-glucuronidase, α-glucosidase, β-glucosidase, N-acetyl-β-glucosaminidase, trypsin, and α-chymotrypsin; and negative for α-mannosidase and α-fucosidase.

The DNA G+C content of the type strain is 46.7 mol% and the genome size is 6.40 Mb. The type strain, C3-R-101^T^ (= KCTC 25440^T^ = CGMCC 1.17981^T^), was isolated from human faeces. The GenBank accession numbers for the 16S rRNA gene sequence and the whole-genome sequence are OK148016 and JAMOKP000000000, respectively.

## Supplemental Materials

Supplementary data for this paper are available on-line only at http://jmb.or.kr.



## Figures and Tables

**Table 1:** Differential phenotypic characteristics of strains M3-R-103^T^ and C3-R-101^T^ and closely related type strains. Strains: 1, M3-R-103^T^; 2, C3-R-101^T^; 3, *Bifidobacterium pseudocatenulatum* DSM 20438^T^; 4, *Blautia hominis* KB1^T^.

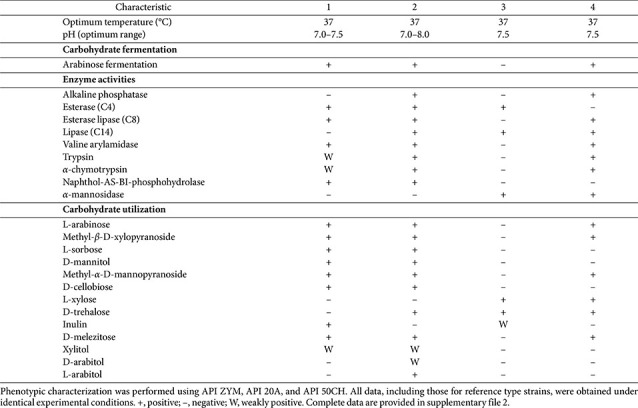

**Table 2:** Analysis of strain M3-R-103^T^ and C3-R-101^T^ resistance to low pH, simulated gastric and intestinal fluids.

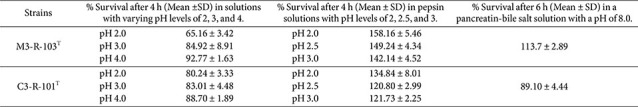

**Table 3:** Bile salt tolerance of strain M3-R-103^T^ and C3-R-101^T^.

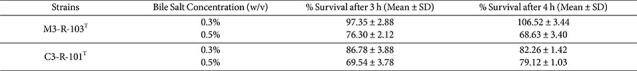

**Table 4:** *In vitro* antioxidant, antidiabetic, and anti-inflammatory activities of M3-R-103^T^ and C3-R-101^T^ in comparison to respective standard drug ascorbic acid, sitagliptin and aspirin, respectively.