# Supplementary material for: Bifidobacterium intestinale sp. nov., and Blautia caeci sp. nov., from the Human Gut Microbiome with Candidate Probiotic Potential
Source: J Microbiol Biotechnol. 2026 May 25;36:e2603013. doi: 10.4014/jmb.2603.03013 (PMC13215868; doi:10.4014/jmb.2603.03013)
Supplement: Supplementary file 1 [file jmb-36-e2603013-supple.pdf]

J. Microbiol. Biotechnol. 2026. 36: e2603013

https://doi.org/10.4014/jmb.2603.03013

Bifidobacterium intestinale sp. nov., and *Blautia caeci* sp. nov., from the human gut microbiome with candidate probiotic potential

Md Shamsuzzaman[1,2], Ram Hari Dahal[2] and Jungmin Kim[1,2*]

[1]Untreatable Infectious Disease Institute, Kyungpook National University, Daegu, Republic of Korea

[2]Department of Microbiology, School of Medicine, Kyungpook National University, Daegu, Republic of Korea

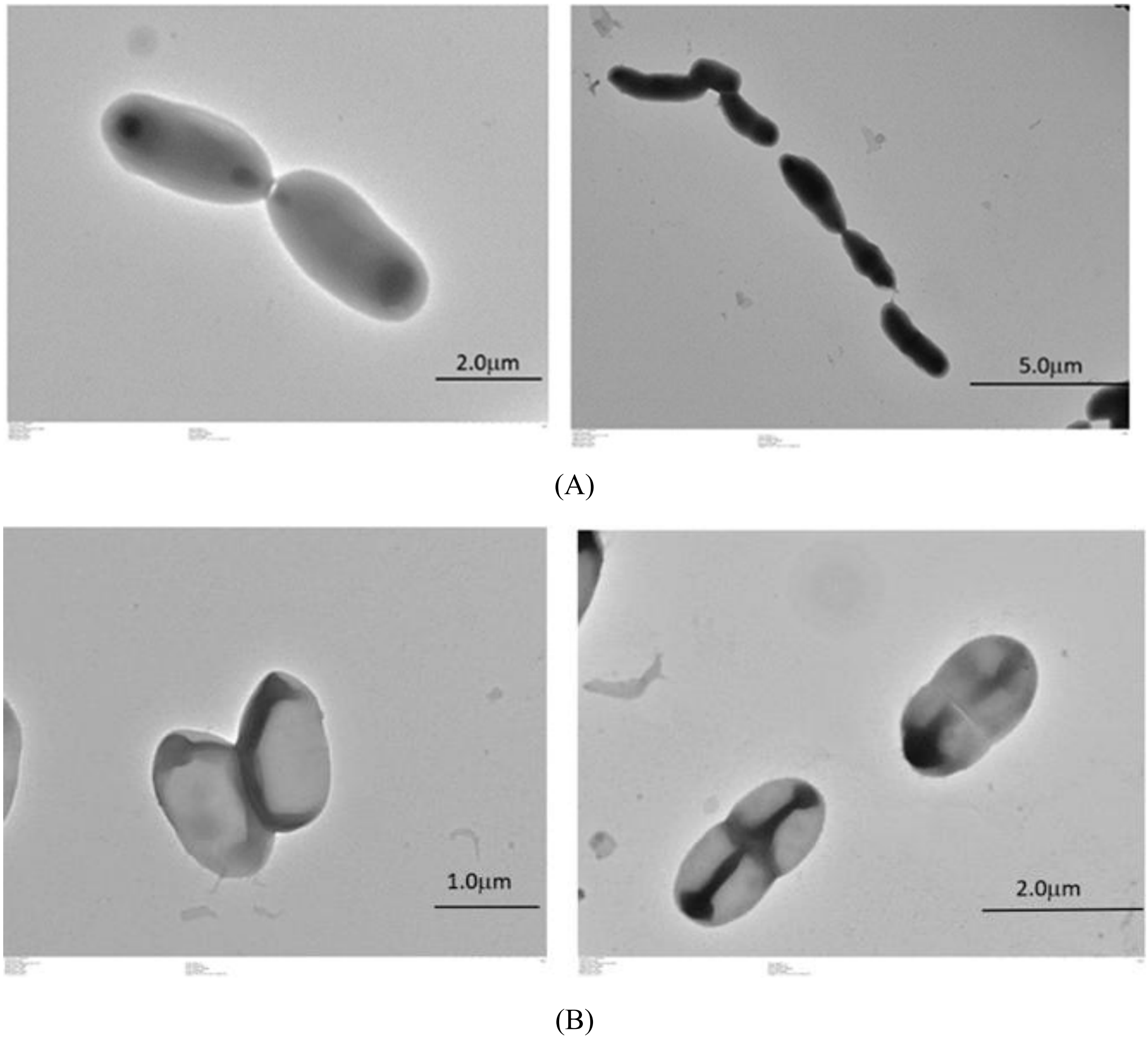

Fig. S1. Transmission electron microscopy images of the novel strains (A) M3-R-103[T] and (B) C3-R-101[T]. Cells were grown on brain heart infusion agar supplemented with 5% sheep blood and incubated anaerobically at 37°C for 24–48 h prior to imaging.

Supplementary

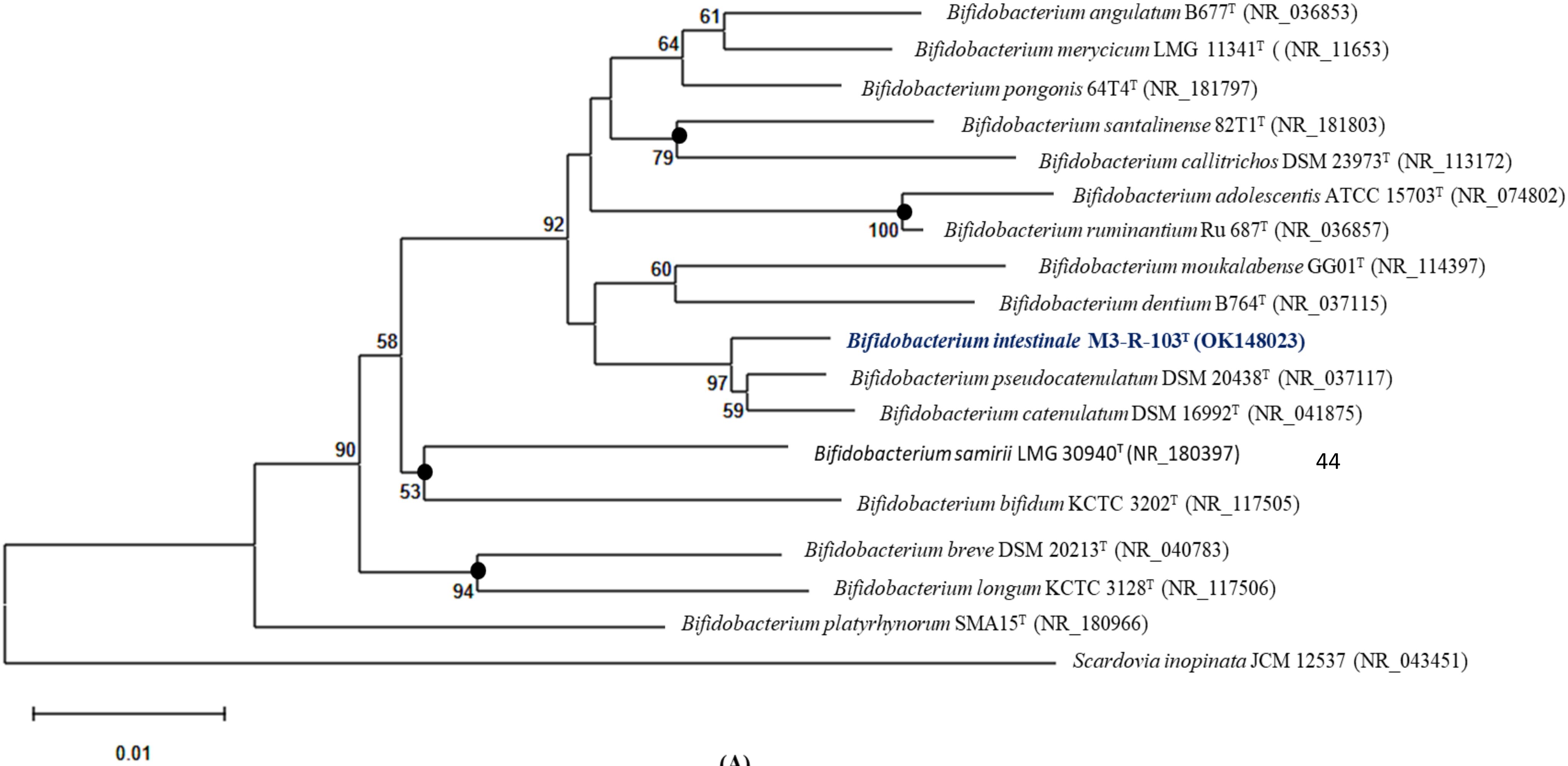

(A)

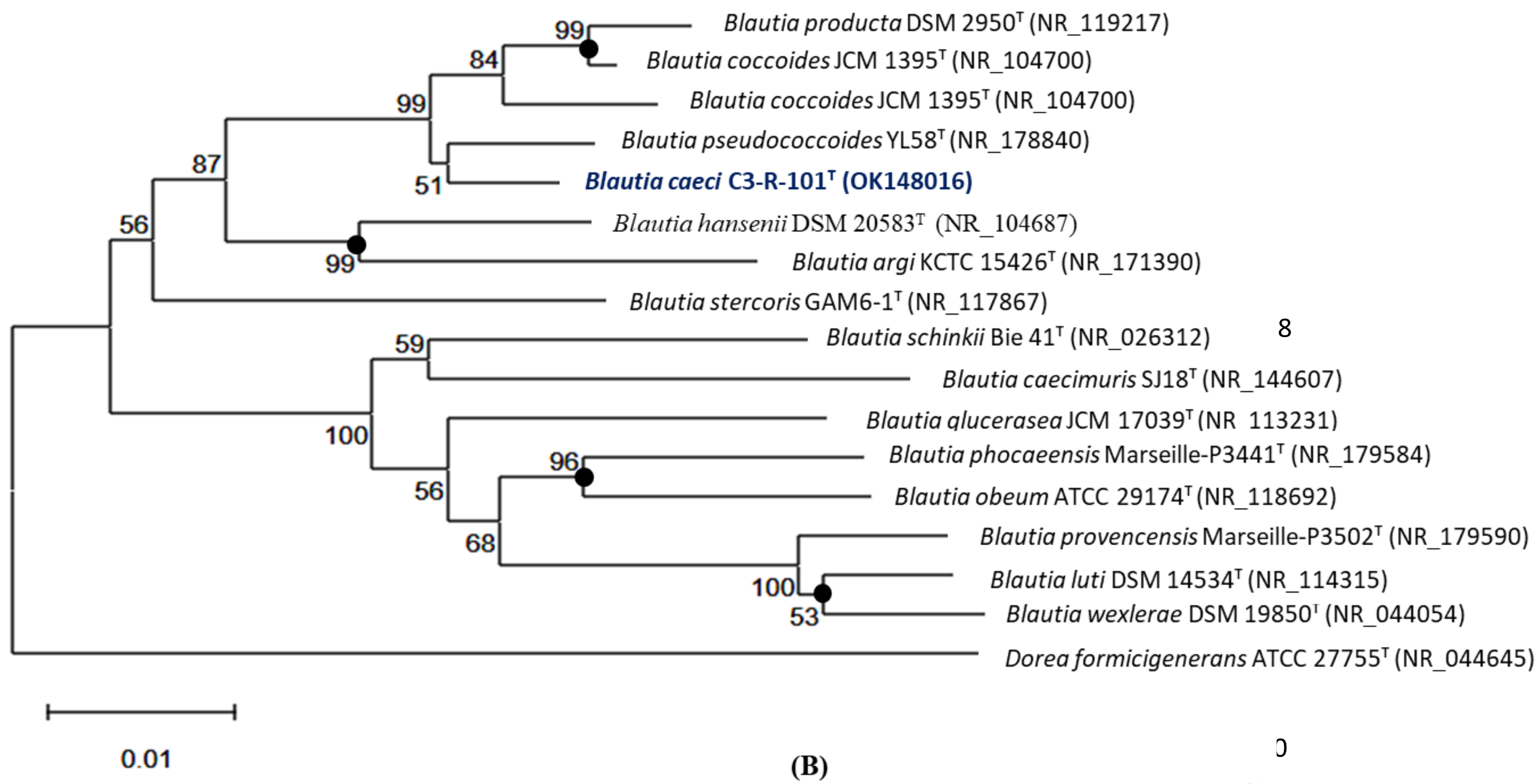

(B)

Fig. S2. The neighbour-joining (NJ) phylogenetic trees based on nearly complete 16S rRNA gene sequences showing the taxonomic positions of strains M3-R-103^{T} and C3-R-101^{T} within the genera *Bifidobacterium* (A) and *Blautia* (B), respectively. Bootstrap values based on 1000 replicates are shown at branch nodes, with only values ≥50% indicated. Nodes consistently recovered by neighbor-joining, maximum-likelihood, and maximum-parsimony methods are marked with filled circles. *Scardovia inopinata* JCM 12537^{T} (NR_043451) and *Dorea formicigenerans* ATCC 27755^{T} (NR_044645) were used as outgroup taxa for panels A and B, respectively. Nucleotide sequence accession numbers are given in parentheses. The scale bar represents 0.01 substitutions per nucleotide position.

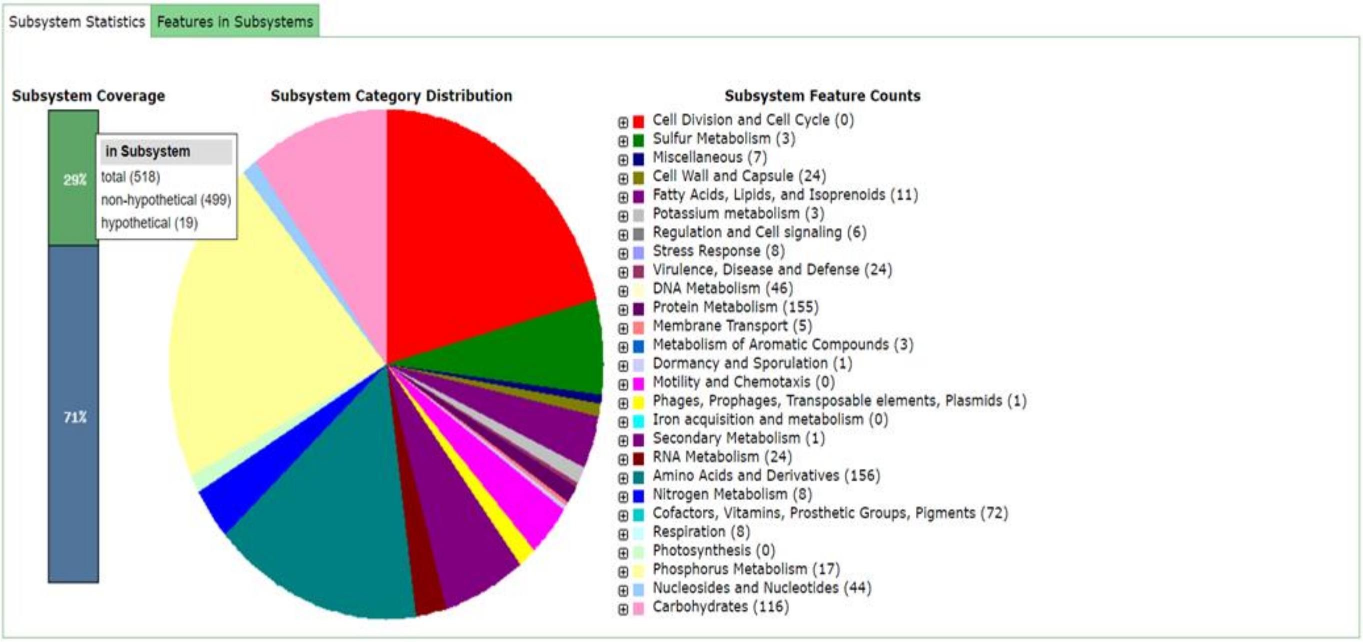

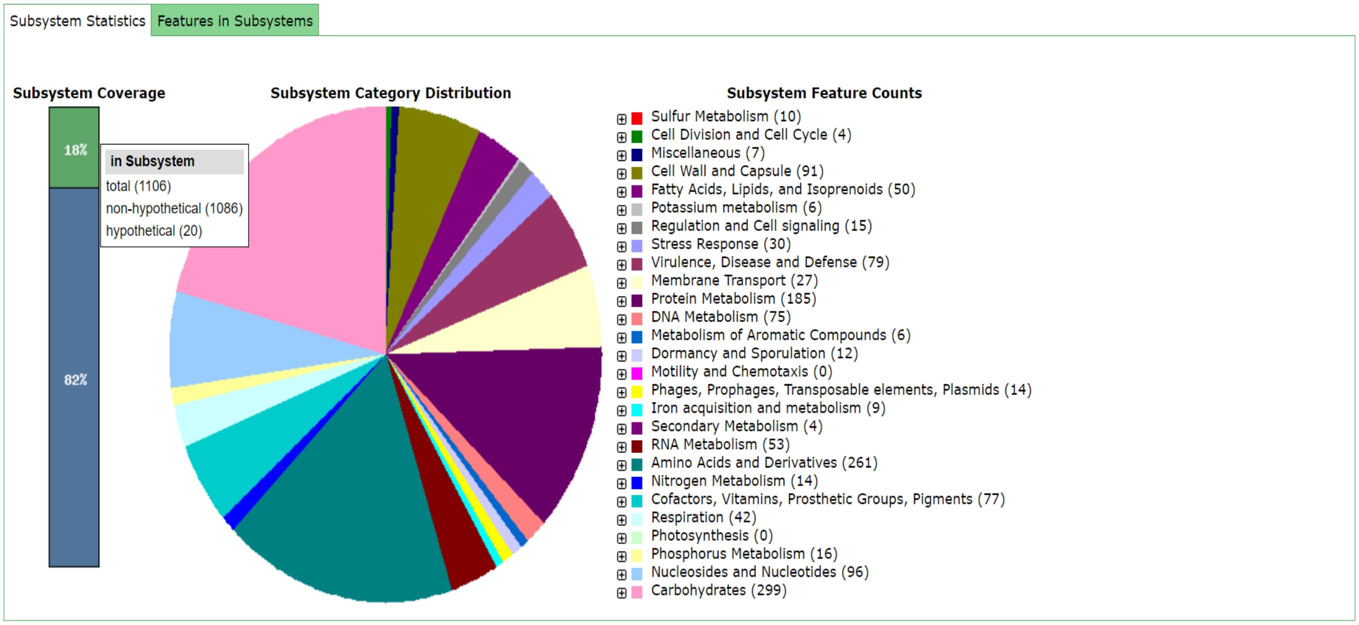

Fig. S3. The subsystem feature counts for strains M3-R-103^{T} and C3-R-101^{T} analysed by RAST (Rapid Annotation using Subsystem technology).

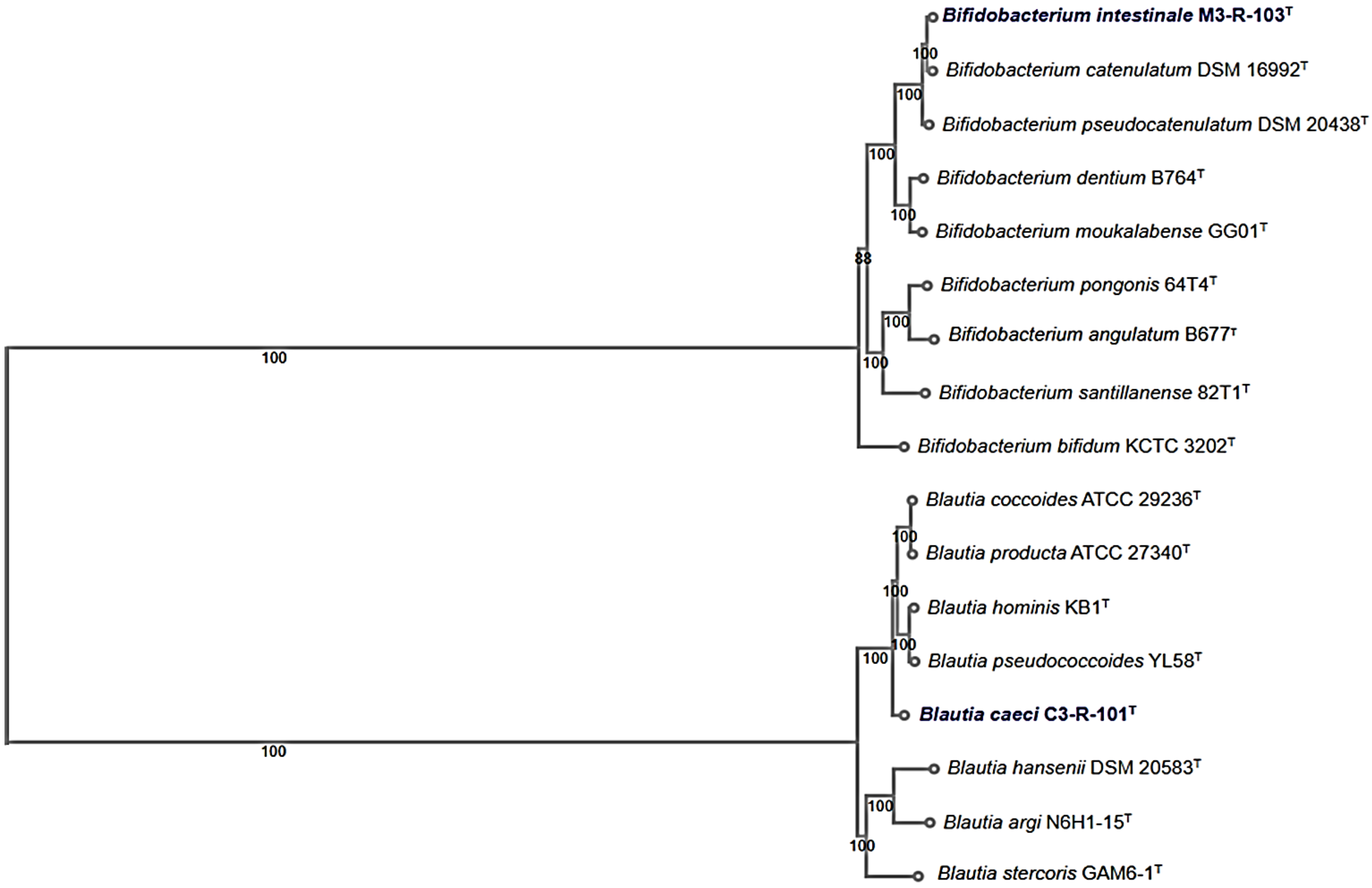
Bifidobacterium intestinale M3-R-103T
Bifidobacterium catenulatum DSM 16992T
Bifidobacterium pseudocatenulatum DSM 20438T
Bifidobacterium dentium B764T
Bifidobacterium moukalabense GG01T
Bifidobacterium pongonis 64T4T
Bifidobacterium angulatum B677T
Bifidobacterium santillanense 82T1T
Bifidobacterium bifidum KCTC 3202T
Blautia coccoides ATCC 29236T
Blautia producta ATCC 27340T
Blautia hominis KB1T
Blautia pseudococcoides YL58T
Blautia caeci C3-R-101T
Blautia hansenii DSM 20583T
Blautia argi N6H1-15T
Blautia stercoris GAM6-1T
100
88

75

(A)

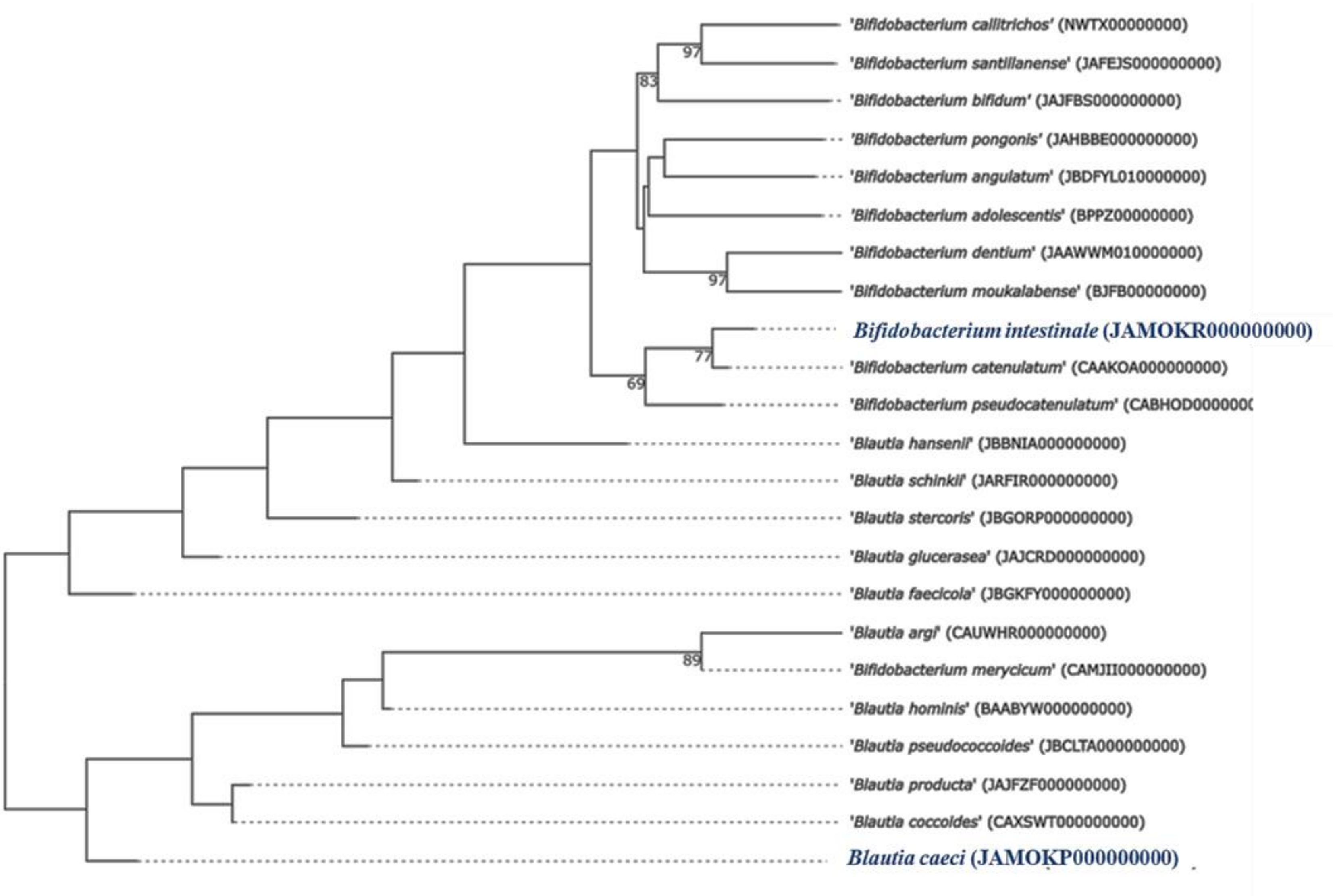

(B)

Fig. S4. Phylogenomic trees showing the taxonomic positions of strains M3-R-103^{T} and C3-R-101^{T}. (A) Maximum likelihood tree reconstructed using RAxML based on concatenated alignments of 100 single-copy core genes. Bootstrap values are shown at branch nodes. **(B)** Genome-based

phylogenomic tree generated using TYGS.Strain M3-R-103^{T} clusters within the genus *Bifidobacterium*, whereas strain C3-R-101^{T} clusters within the genus *Blautia*. Type strains are indicated by a superscript T. Trees were visualized using iTOL. The scale bar represents substitutions per site.

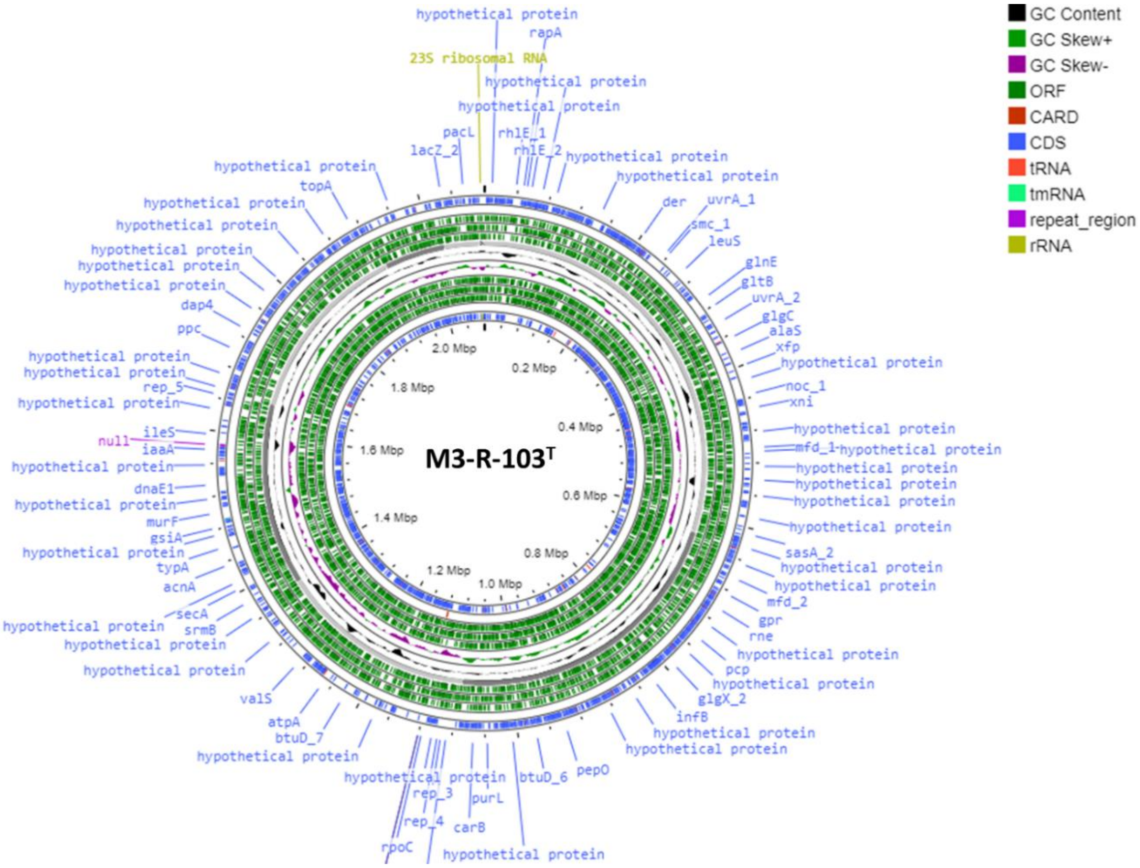

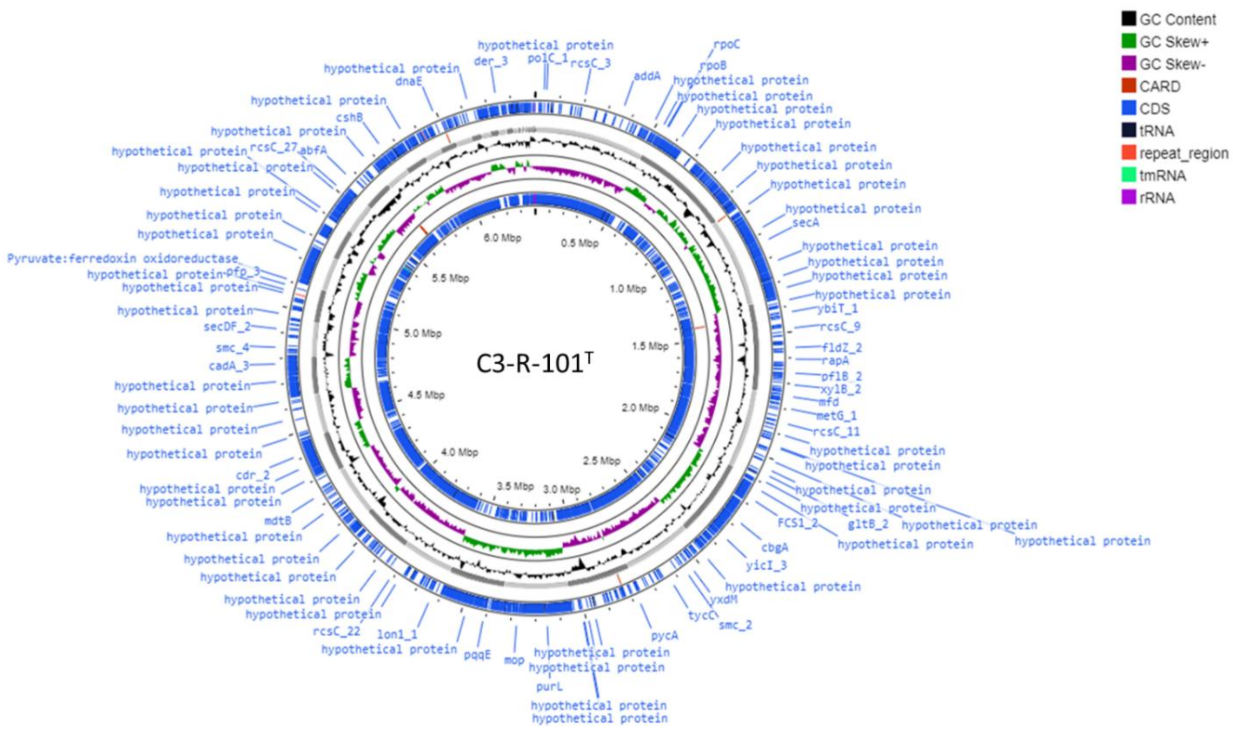

Fig. S5. The figure shows a graphical circular representation of three chromosomes of strains M3-R-103^{T} and C3-R-101^{T}. The figure displays various genomic features such as CDS on the forward and reverses strands, tRNA, rRNA, GC content, and GC skew, marked from the edge to the center.

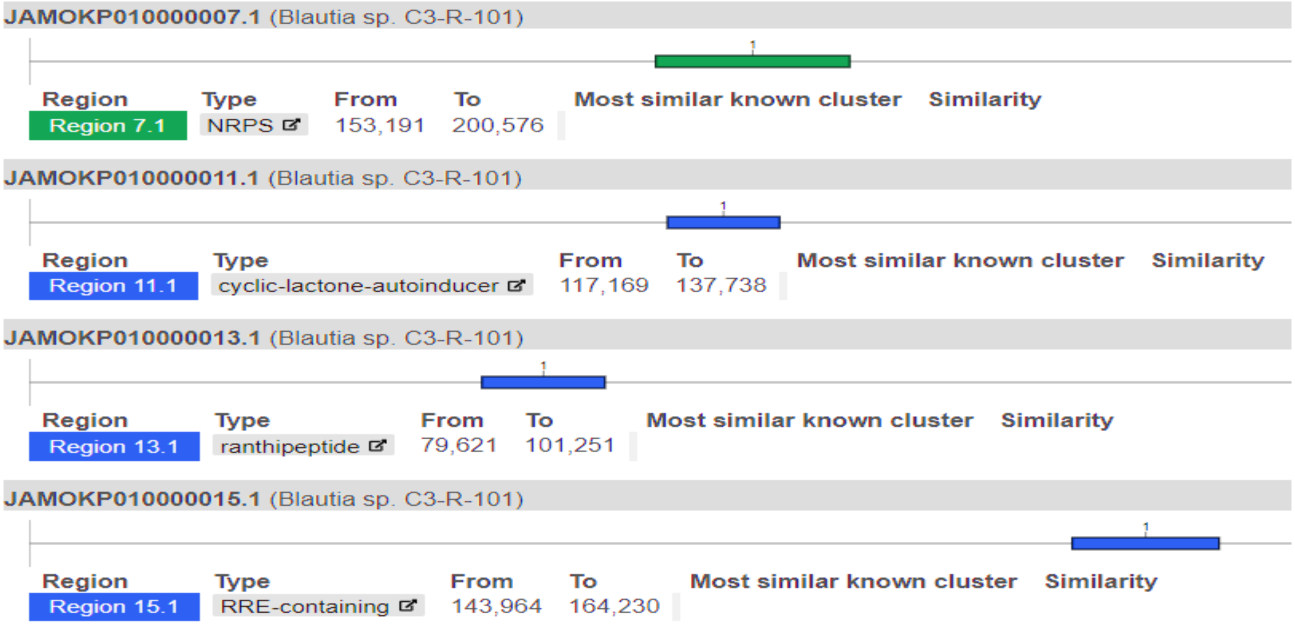

JAMOKP010000007.1 (Blautia sp. C3-R-101)

Region	Type	From	To	Most similar known cluster	Similarity
Region 7.1	NRPS	153,191	200,576		

JAMOKP010000011.1 (Blautia sp. C3-R-101)

Region	Type	From	To	Most similar known cluster	Similarity
Region 11.1	cyclic-lactone-autoinducer	117,169	137,738		

JAMOKP010000013.1 (Blautia sp. C3-R-101)

Region	Type	From	To	Most similar known cluster	Similarity
Region 13.1	ranthipeptide	79,621	101,251		

JAMOKP010000015.1 (Blautia sp. C3-R-101)

Region	Type	From	To	Most similar known cluster	Similarity
Region 15.1	RRE-containing	143,964	164,230		

Fig. S6. Predicted biosynthetic gene cluster for secondary metabolites in strains C3-R-101^{T}.

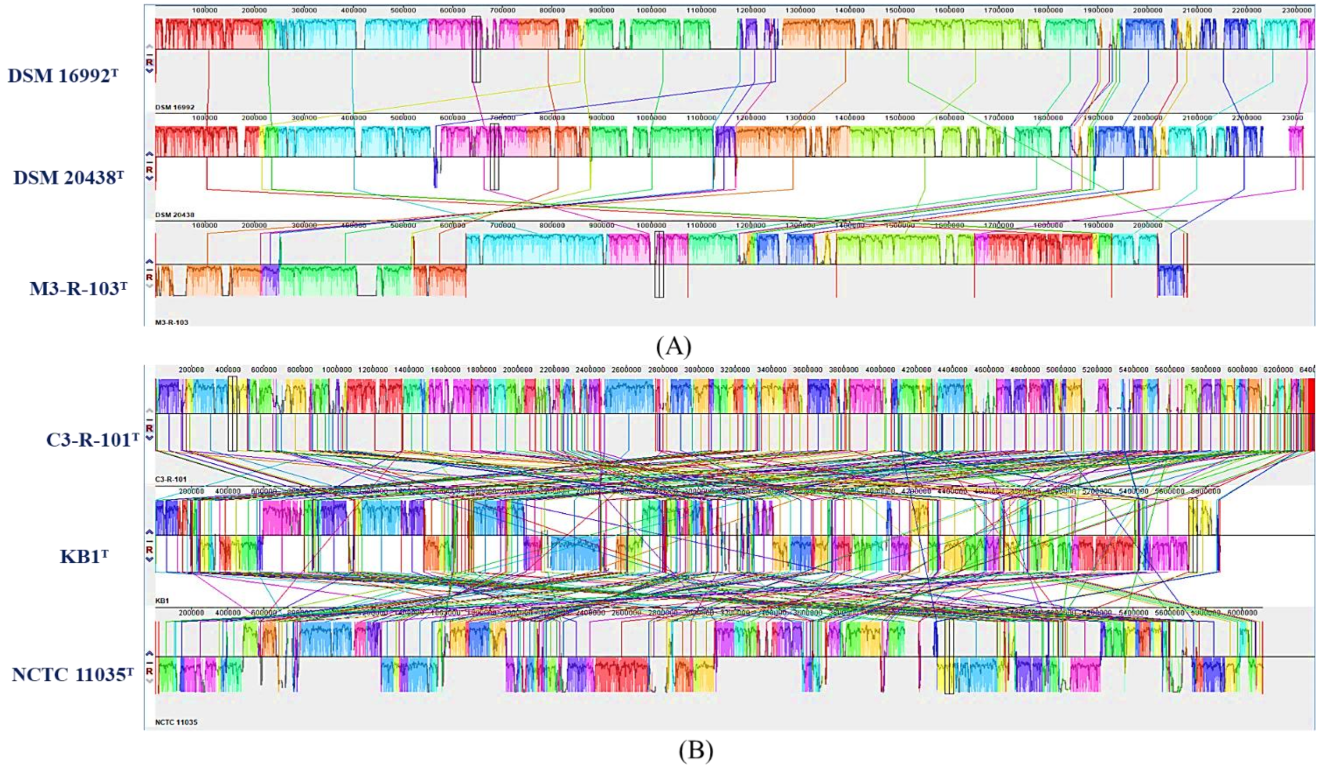

Fig. S7. Whole-genome alignments generated using progressive Mauve (v2.3.1) showing genome-wide synteny among strains M3-R-103ᵀ and C3-R-101ᵀ and their closest relatives. Homologous regions are represented as locally collinear blocks (LCBs), with inversions shown below the center line. White regions indicate strain-specific sequences. The upper panel (*Bifidobacterium*) shows largely conserved synteny, whereas the lower panel (*Blautia*) reveals greater structural variation, including rearrangements and inversions, supporting genomic divergence between strains.

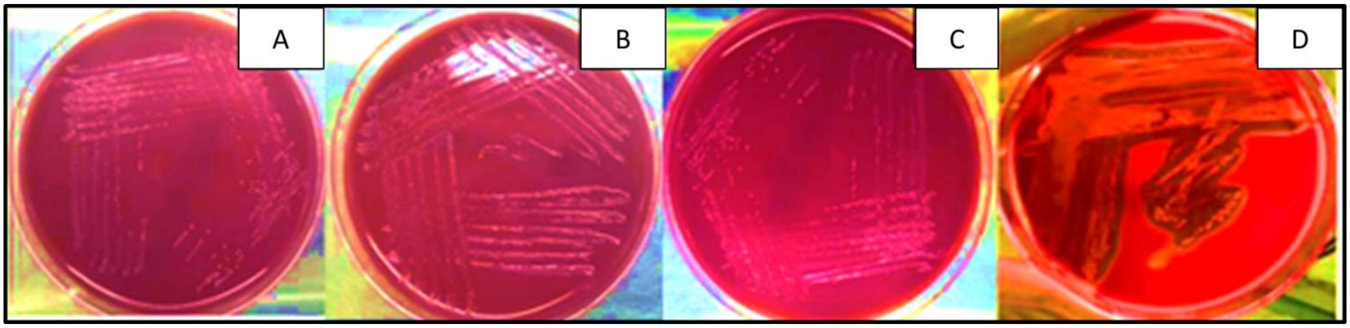

Figure S8. Hemolytic activity assay of strains M3-R-103ᵀ and C3-R-101ᵀ. Hemolysis was assessed on blood agar after anaerobic incubation at 37°C for 48 h. (A) M3-R-103ᵀ, (B) C3-R-101ᵀ, (C) *Segatella intestinalis* B2-R-102ᵀ (non-hemolytic negative control), and (D) *Cutibacterium acnes* KB112 (hemolytic positive control).

Table S1. 16S rRNA gene sequence similarities (%) of strains M3-R-103^{T}, and C3-R-101^{T} against closest members of the genus *Bifidobacterium and Blautia*.

Bifidobacterium	**NCBI (%)**	**EzBioCloud (%)**
Bifidobacterium intestinale M3-R-103^{T}	-	-
Bifidobacterium pseudocatenulatum DSM 20438^{T}	98.9	99.1
Bifidobacterium catenulatum DSM 16992^{T}	98.8	98.8
Bifidobacterium angulatum B677^{T}	97.2	97
Bifidobacterium moukalabense GG01^{T}	97	97.3
Bifidobacterium pongonis 64T4^{T}	97.5	97.4
Bifidobacterium santillanense 82T1^{T}	96.4	96.5
Bifidobacterium dentium B764^{T}	96.2	96.3
Bifidobacterium bifidum KCTC 3202^{T}	96	95.9
Blautia	NCBI (%)	EzBioCloud (%)
Blautia caeci C3-R-101^{T}	-	-
Blautia hominis KB1^{T}	98.6	98.8
Blautia coccoides ATCC 29236^{T}	98.3	98.3
Blautia pseudococcoides YL58^{T}	98.2	98.2
Blautia producta ATCC27340^{T}	98	98.6
Blautia hansenii DSM 20583^{T}	96.4	96.3
Blautia argi N6H1-15^{T}	95.3	95.1
Blautia stercoris GAM6-1^{T}	96.3	96.1

Table S2. Genomic features of strains M3-R-103^{T}, and C3-R-101^{T}.

**Attributes**	**Features**	
	**M3-R-103^{T}**	**C3-R-101^{T}**
Accessions	JAMOKR000000000	JAMOKP000000000
Genome size (bp)	2,080,619	6,401,851
G+C content (mol%)	56.3	46.7
No of contigs	9	58
N50	448464	289867
Total genes	1,741	5,868
CDSs (Total)	1,677	5,792
Number of Coding Sequences	1792	6223
Genes (RNA)	61	65
rRNAs (5s,16s, 23S)	4,1,1	1,3,7
tRNAs	55	61
ncRNAs	3	4

Pseudo genes	23	101
Number of Subsystems	196	259
Genome coverage	150.0x	150.0x
Resistances gene (Card)	-	*ermX*, *VanHDX*, *msrD*, and *tet*(o)

Table S3. ANI and dDDH (%) values of strains M3-R-103^T, and C3-R-101^T against closest members of the genus *Bifidobacterium and Blautia*.

Bifidobacterium	**ANI (%)**	**dDDH (%)**
Bifidobacterium intestinale M3-R-103^T	-	-
Bifidobacterium pseudocatenulatum DSM 20438^T	92.1	44.9
Bifidobacterium catenulatum DSM 16992^T	93.6	54.5
Bifidobacterium angulatum B677^T	77.8	25.5
Bifidobacterium moukalabense GG01^T	80.2	24.4
Bifidobacterium pongonis 64T4^T	77.7	26.2
Bifidobacterium santillanense 82T1^T	78.28	23.7
Bifidobacterium dentium B764^T	80.2	24.1
Bifidobacterium bifidum KCTC 3202^T	76.5	24.3
Blautia	**ANI (%)**	**dDDH (%)**
Blautia caeci C3-R-101^T	-	-
Blautia hominis KB1^T	83.9	29.6
Blautia coccoides ATCC 29236^T	84.8	30.0
Blautia pseudococcoides YL58^T	84.8	29.9
Blautia producta ATCC27340^T	84.6	29.7
Blautia hansenii DSM 20583^T	72.8	22.8
Blautia argi N6H1-15^T	73.7	19.6
Blautia stercoris GAM6-1^T	73.2	22.9

Table S4. Percentage inhibition of growth of various pathogens by M3-R-103^{T} and C3-R-101^{T} culture supernatants.

Name of the pathogens	% inhibition	
	**M3-R-103^{T}**	**C3-R-101^{T}**
E. coli ATCC 25922	-27.73±0.36	-22.86±6.63
Acinetobacter baumannii ATCC 17978	-36.44±23.76	52.82±15.12
S. felis ATCC 49168	65.37±1.10	63.37±0.11
Acinetobacter junii strain 16	70.41±7.84	77.09±4.81
S. pseudointermedius ATCC 49051	39.33±3.00	45.53±2.30
S. edidermidis ATCC 1228	43.46±1.71	52.67±0.17
S. schleferi ATCC 43808	16.39±3.39	26.73±2.59
S. capre KCTC 3583	6.20±3.36	21.06±1.26
Salmonella enteritidis PT4	17.15±2.53	18.55±2.18

Table S5. ***In vitro* antibiotic susceptibility profiles of strains M3-R-103^{T} and C3-R-101^{T}.**

Antibiotic (Class)	M3-R-103^{T} MIC (µg/mL)	C3-R-101^{T} MIC (µg/mL)
Cefoxitin (Cephamycin)	≤2	≤4
Ampicillin (Penicillin)	≤0.125	≤0.125
Piperacillin/Tazobactam (*β*-lactam/*β*-lactamase inhibitor)	≤2	≤4
Imipenem (Carbapenem)	≤1	≤0.5
Erythromycin (Macrolide)	≤0.25	1
Tetracycline (Tetracycline)	≤2	≥16
Vancomycin (Glycopeptide)	≥16	≥16

MICs were determined by broth microdilution according to CLSI M11 (9th edition) guidelines for anaerobic bacteria. MIC was defined as the lowest concentration inhibiting visible growth after 48 h of anaerobic incubation at 37°C. Values are reported without categorical interpretation where genus-specific breakpoints are unavailable.
